# Laser-driven proton acceleration from ultrathin foils with nanoholes

**DOI:** 10.1038/s41598-021-84264-z

**Published:** 2021-03-03

**Authors:** Giada Cantono, Alexander Permogorov, Julien Ferri, Evgeniya Smetanina, Alexandre Dmitriev, Anders Persson, Tünde Fülöp, Claes-Göran Wahlström

**Affiliations:** 1 Department of Physics, Lund University, SE-22100 Lund, Sweden; 2 Department of Physics, Chalmers University of Technology, SE-41296 Göteborg, Sweden; 3 Department of Physics, University of Gothenburg, SE-41296 Göteborg, Sweden

**Keywords:** Physics, Plasma physics, Laser-produced plasmas

## Abstract

Structured solid targets are widely investigated to increase the energy absorption of high-power laser pulses so as to achieve efficient ion acceleration. Here we report the first experimental study of the maximum energy of proton beams accelerated from sub-micrometric foils perforated with holes of nanometric size. By showing the lack of energy enhancement in comparison to standard flat foils, our results suggest that the high contrast routinely achieved with a double plasma mirror does not prevent damaging of the nanostructures prior to the main interaction. Particle-in-cell simulations support that even a short scale length plasma, formed in the last hundreds of femtoseconds before the peak of an ultrashort laser pulse, fills the holes and hinders enhanced electron heating. Our findings reinforce the need for improved laser contrast, as well as for accurate control and diagnostics of on-target plasma formation.

## Introduction

Starting from twenty years ago, laser-driven ion acceleration has been the object of tireless efforts of theoretical understanding and experimental optimisation, stimulated by the remarkable beam properties (in terms of duration, brightness, emittance) that promote the use of such sources in different applications (e.g. time-resolved radiography, isochoric heating and probing of warm dense matter, hadron therapy and PET isotope generation, fundamental studies of plasma and nuclear physics)^1–3^. To attain the beam quality required for each application, various acceleration mechanisms are currently being explored, developed hand in hand with laser and target technology, beam transport and diagnostics. Target normal sheath acceleration (TNSA) is up to now the most practised mechanism to generate proton and ion beams with tens of MeV of energy. In this process, a high-power laser pulse accelerates electrons at the front surface of thin, solid targets. Once some hot electrons travel through the target and escape into vacuum, the resulting space-charge electric field ionises atoms and hydrocarbon impurities on the rear surface, accelerating protons and heavier ions perpendicularly to it.

Increasing the energy transfer from the laser pulse to the hot electrons that develop the accelerating sheath field (i.e. increasing the absorption) results in larger yields and energies for the TNSA-driven ion beams. To this aim, a well-established strategy consists of adding micro- and nanostructures on the front surface of the target^4^. In the past decade, advances in the target manufacturing techniques and in the control of the laser performances have enabled experiments on various types of nanostructures^5^, such as micropillars^6^, nanowires^7^, nanospheres^8^, foams^9,10^, or gratings^11,12^. At the same time, numerical simulations have explored how periodic arrangements of different shapes, sizes and geometries affect the mechanisms of electron heating^13–21^, for example by providing larger areas for the laser to accelerate the plasma electrons, or by favouring charge recirculation and the occurrence of resonant processes^9–12^. Experiments with nanostructured targets have reported factors between 1.2 and 2.5 for the enhancement of the maximum proton energy with respect to simple flat foils. Faced with similar laser-to-proton energy conversion efficiencies, crucial aspects in the choice of the most efficient nanostructures become the costs of manufacturing and handling, which in turn depend on how stringent the geometrical constraints are^7,11,22^.

In this context, we investigated the maximum energy of proton beams accelerated from flat foils of gold, perforated with a non-periodic distribution of nanometric holes (nanoholes, NHs). Previous experimental works on porous targets, composed of a periodic array of several-$\upmu$m-long nanochannels, reported an increase of the X-ray yield when irradiating at sub-relativistic intensities (i.e. below $10^{18}$ W/cm$^2$)^23,24^. The use of perforated foils of sub-micrometric thicknesses to optimise laser-driven proton acceleration, however, has been explored only numerically^20,21,25,26^, and to our knowledge no experiments in the relativistic regime have been performed so far. Particle-In-Cell (PIC) simulations showed that plasma electrons extracted from the edges and from the walls of the NHs locally amplify the electromagnetic field at the vacuum-plasma boundary. Electron experiencing this field gain more energy compared to non-structured foils, as they also benefit from a longer acceleration time due to longitudinal and transverse recirculation across the dense regions surrounding the holes. In a recent numerical study^26^, these effects were related to the presence of individual holes on the target surface, rather than to their spatial distribution or to the reduced mass of the foil. With a greater number of highly energetic electrons crossing the target, enhancement factors of the maximum proton energy between 1.4 and 2 were reported for a variety of NH parameters^26^.

From an experimental point of view, the relaxation in the geometrical constraints of the nanostructures reduces the technical difficulties associated with target fabrication. In this work, NH targets were produced with hole-mask colloidal lithography^27^, a method where a metallic film is deposited on a distribution of plastic nanospheres of a chosen diameter. The nanospheres are then stripped away, leaving holes in the flat foil. Since the structuring does not include brittle elements such as nanospheres or nanowires, these foils can be efficiently produced in large areas and easily mounted on different supports to fit the experimental setup. For the same reason, the foil can be reduced to sub-$\upmu$m thicknesses, which usually also favours proton acceleration^28,29^.

**Figure 1:**
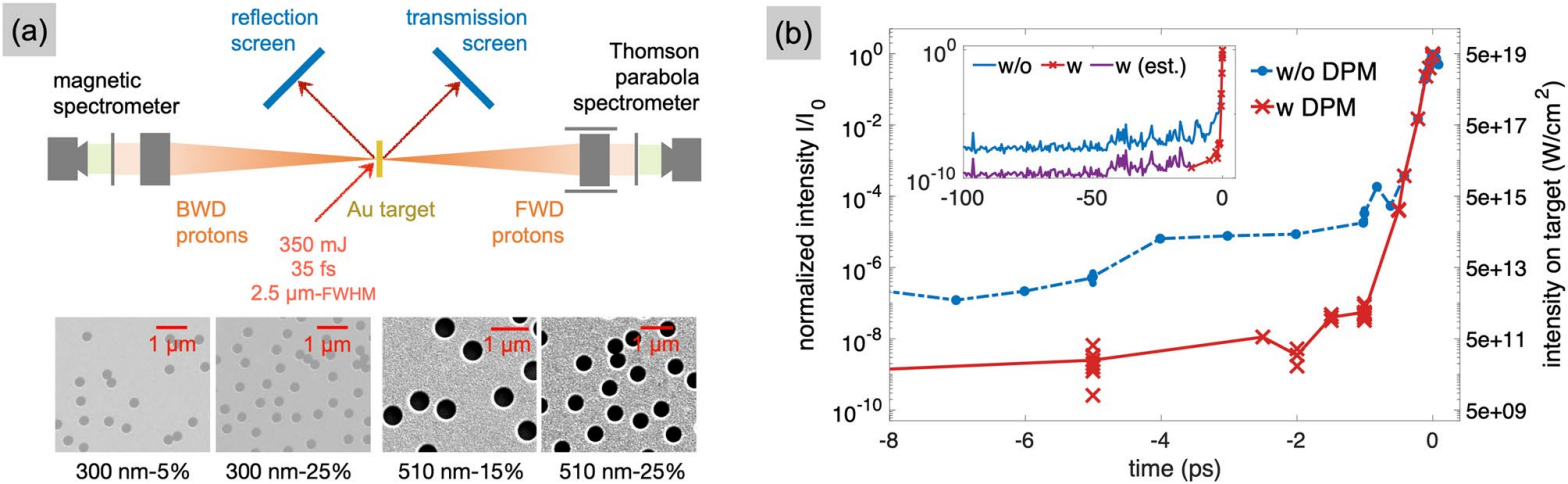
(**a**) Layout of the experimental setup and images of NH targets. Indicated are the NH diameter and filling factors. See Methods for additional information. (**b**) Laser temporal contrast measured by a third-order cross-correlator, with and without DPM (crosses and dots, respectively). The lines connect the averages of the points measured at each time. In the inset, the measurement without DPM is extended to include the ASE pedestal. The darker section of the curve with DPM is an estimation of the contrast where the signal falls below the dynamic range of the cross-correlator.

On the other hand, it is well known that exposing ultrathin and nanostructured targets to relativistic intensities requires lowering the level of amplified spontaneous emission (ASE) and pre-pulses inherent to chirped pulse amplification-based laser systems. This would otherwise cause early heating, deformation of the target surface or, in the worst case, the creation of a plasma above critical density ($n_c = \epsilon _0 m_e \omega ^2/e^2$, $\omega$ being the laser frequency). Increasing the laser temporal contrast (i.e. the ratio between the peak intensity of the ultrashort pulse and any other radiation prior to it) so that the intensity of the ASE pedestal and of any pre-pulse is below the ionisation threshold of the target material is thus a prerequisite for this kind of experiments^30,31^. The High Power Laser Facility of Lund University (Sweden), where this work has been performed, houses a double plasma mirror (DPM)^31,32^ that reduces at least by $\sim 2$ orders of magnitude the intensity of the radiation before the ultrashort pulse (cf. Fig. 1b). In this way, we achieve a temporal contrast of $10^{10}$ at the ASE timescale, and of $10^{8}$ at 2 ps before the ultrashort pulse.

Our experiments aimed at testing, for the first time, the efficiency of NH targets irradiated by intense, high contrast laser pulses. However, despite varying the target parameters, the maximum energy of TNSA-driven protons was found to be equivalent to the one measured from flat foils of comparable thickness. We support our experimental results with new PIC simulations, where we investigated early plasma formation due to the finite contrast of our laser pulse. It appears that the energy deposited on target in the last ps before the peak of the pulse, when the DPM is ineffective, is enough to create a plasma that fills the NHs, thus hampering the expected enhancement in electron heating.

## Results

Protons were accelerated irradiating flat foils of gold, both with and without NHs, with a multi-TW, high contrast laser pulse. We tested NH targets with various hole diameter *d*, filling factor $\rho$ (i.e. the area of the foil covered by the holes, per unit area) and foil thickness *t*. Under identical conditions, we always performed measurements also from foils of the same thickness, but without NHs. These latter will be referred to as *regular* foils in the remainder of the text.

Figure 1 illustrates the experimental setup, including the laser focused at $45^\circ$ angle of incidence, two diffuse reflectance screens for measuring the reflected and transmitted laser light during the interaction, and the diagnostics for protons emitted in both forward (FWD) and backward (BWD) directions.

Observing the cutoff energies of the protons accelerated in the BWD direction is a standard procedure to ensure that the laser contrast is high enough to prevent plasma formation on the target surface, as a step-like density profile enables efficient laser absorption through the vacuum-heating mechanism^33,34^ and the establishment of the TNSA-driving fields also on the front side of the target. With good enough contrast, foils with sub-$\upmu$m thicknesses typically produce similar proton energies in both FWD and BWD directions, giving an indirect evidence of the survival of the target prior to the interaction with the pulse peak^35^. In our experiment, preliminary shots on the thinnest regular foils (120 nm of thickness) resulted in a maximum FWD energy of $(6.7 \pm 0.2)$ MeV, while the maximum BWD energy was $(6.7 \pm 0.7)$ MeV. As a consequence, and since in the fabrication of the NH targets the diameter of the holes was always larger than the foil thickness, the target structuring was assumed to be preserved during each laser shot.

**Figure 2:**
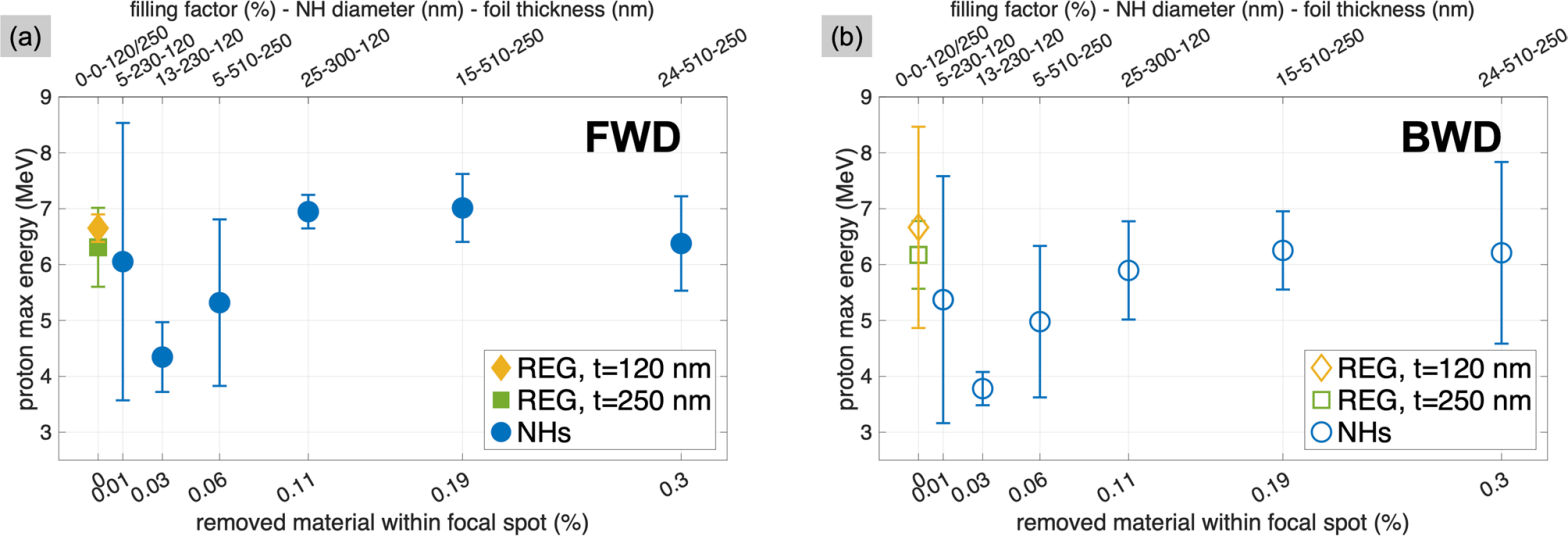
Proton maximum energies obtained from NH targets and regular foils, both in the FWD (**a**) and BWD (**b**) direction. For NHs, the amount of removed material is defined by the area occupied by NHs within the area covered by the laser focal spot. All target parameters (filling factor $\rho$, NH diameter *d*, foil thickness *t*) are listed in the top x-axis.

The maximum energies of the protons accelerated from all the different targets, both in the FWD and BWD direction, are presented in Fig. 2. At a first glance, similar values and trends in both directions indicate efficient proton acceleration with all the types of targets, with the maximum energies in the FWD direction exceeding those in the BWD direction by less than $20\%$. However, more importantly, the results also show that the NH targets do not produce any proton energy enhancement, no matter the parameter we varied in our scan: The maximum energies are always found to be comparable with the values obtained from regular foils.

**Figure 3:**
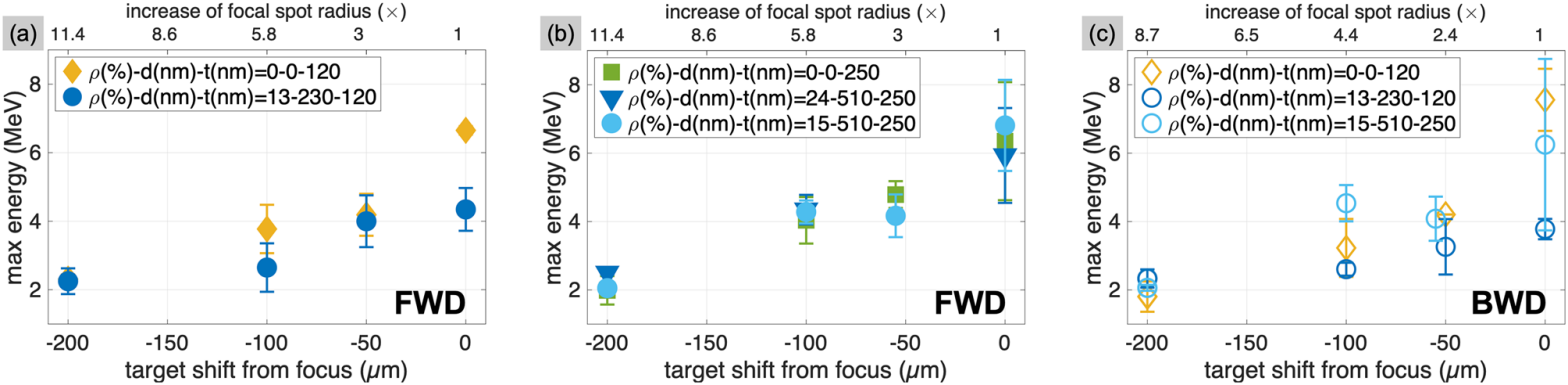
Proton maximum energies measured from regular and perforated foils when increasing the illuminated surface of the target. The increase of the focal spot radius is calculated as $[1+(z/z_\text {R})^2]^{0.5}$, with *z* being the shift from focus and $z_\text {R}$ the Rayleigh length determined in the experiment. (**a**) FWD direction, for targets of 120 nm thickness. (**b**) FWD direction, for targets of 250 nm thickness. (**c**) BWD direction, for a selection of targets.

In NH targets with small filling factors, the number of holes inside the area covered by the laser focal spot varies relatively more the smaller the filling factor is. In order to ensure that NHs were irradiated, we hence varied the area covered by the focal spot by shifting the target along the focal axis. The consequent decrease of the laser intensity, up to a factor of $\sim 100$, would also limit any damage of the target possibly occurring in the residual time between the triggering of the DPM and the arrival of the ultrashort pulse. However, the results reported in Fig. 3 indicate once again that the maximum energy from NH targets never exceeds that from the regular foils, and the energies in both FWD and BWD direction remain comparable regardless of the target type or the position along the focal axis.

**Figure 4:**
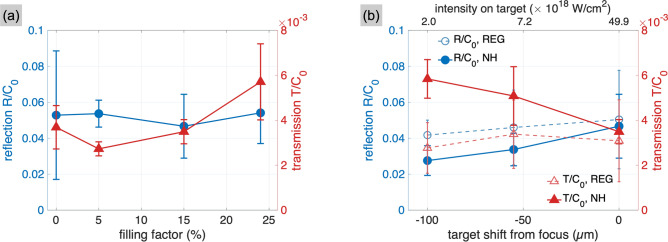
Reflection (dots) and transmission (triangles) signals, normalised to the signal $C_0$ measured in transmission without any target (blank shot), as a function of the filling factor (**a**) and of the shift of the target from the focal position (**b**). The focus scan is limited to the points where a sufficient signal to noise ratio could be detected on the reflectance screens. For all the measurements in this figure, the NH diameter was 510 nm and the foil thickness 250 nm. For the NH targets in (**b**), the filling factor was $15\%$.

The results on the proton energies suggest that the NHs have been altered on the nanometric scale before the laser pulse reaches the peak intensity on the target, thus preventing the efficient electron heating that would support the enhanced proton acceleration. In Fig. 4, we present the measurements of the laser reflection and transmission from regular foils and NH targets, obtained with the diffuse reflectance screens. Here it is worth noting that, although displaying a slight increase in transmission for larger NH densities (Fig. 4a), all targets produce similar absorption factors. This disagrees with the numerical simulations performed with a laser pulse with infinite temporal contrast^26^, where a $70\%$ decrease in reflection and $500\%$ increase in transmission were observed when structuring the foil with NHs. A similar effect is observed when displacing the targets out of focus, as shown in Fig. 4b. When reducing the laser intensity on the NH targets, the transmission signal almost doubles compared to when the target is placed at focus, while the the reflection is 10 to $30\%$ lower than the signal from regular foils. Despite hinting at the survival of the holes, the absorption coefficients that derive from the combination of the reflection and transmission measurements differ, for the regular foil and NH target, by only $1\%$.

In a separate set of measurements we changed the laser incidence angle to $20^\circ$ and determined the FWD proton energy from NH targets (having $d=230$ nm, $t=120$ nm and $\rho =15\%$) and from the corresponding regular foil. With observed cutoffs of $(4.1\pm 0.4)$ and $(5.1 \pm 0.6)$ MeV, respectively, we conclude that neither in this case did the presence of NHs result in enhanced proton energies.

Led by the experimental observations, we performed two-dimensional PIC simulations to investigate how a pre-plasma, created in the last 100s of fs before the interaction with the relativistically intense laser pulse, affects the efficacy of NH targets. We characterise the pre-plasma by the scale length *L* of the exponential density profile that describes its expansion^36^.

**Figure 5:**
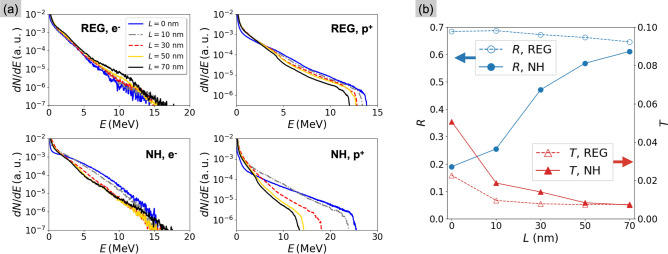
Numerical results from scanning the pre-plasma scale length. (**a**) Energy spectra from from regular foils (top) and NH targets (bottom). Electron spectra (left column) include all electrons recorded in the simulation box at the peak intensity, $t=0$ fs, while proton spectra (right column) include the protons on the rear side of the target at $t=250$ fs after the peak. (**b**) Reflection and transmission coefficients for both target types.

In a first set of simulations, a pre-plasma with an arbitrary scale length was added on the target before the interaction with an infinite-contrast laser pulse. Given the thinness of the foils, the pre-plasma was expanding from both front and rear surfaces, as well as from the walls of the holes in NH targets. A simple estimation for the scale length required to reach the relativistic critical density $\gamma n_c$ at the centre of the holes gives $L_\text {fill}=-d/[2\log (\gamma n_c/2n_{e0})]$, with *d* the NH diameter, $\gamma =(1+a_0^2)^{1/2}$ with $a_0$ the normalised vector potential, and $n_{e0}$ the initial electron density of the target. In these simulations, $d=200$ nm, $n_{e0}\simeq 370 n_c$ and $a_0=6.7$ (i.e. a peak intensity $I_0 \simeq 10^{20}$ W/cm$^2$), resulting in $L_\text {fill} \simeq 20$ nm.

Figure 5a shows the electron and proton spectra from regular foils and NH targets when the pre-plasma scale length varies between 0 and 70 nm. In the case of regular foils (top row), slightly enhanced heating of 10 MeV electrons suggests a transition towards resonant absorption in correspondence of longer pre-plasmas ($L \ge 50$ nm). However, the maximum proton energy progressively decreases with increasing *L*, because the wider density ramp at the rear surface hampers the formation of the accelerating sheath field^37,38^. The results with NH targets (bottom row) indicate a stronger dependence on the scale length. First, increasing the scale length suppresses the large population of electrons between 5 and 12 MeV that is accelerated from the NH walls when $L=0$ nm^26^. Second, in addition to the the gradual decrease as observed with the regular foil, the proton energies show a larger drop, from values around 25 MeV when $L<L_\text {fill}$, to 13 MeV in the opposite case. The effect of the pre-plasma on target absorption is presented in Fig. 5b. While the absorption of the regular foil (dashed lines) is almost constant for $L>10$ nm, the NH target (solid lines) shows a $90\%$ decrease in transmission and a $200\%$ increase in reflection when *L* reaches 70 nm. At this point, absorption is similar for both targets, in agreement with the experimental results of Fig. 4.

**Figure 6:**
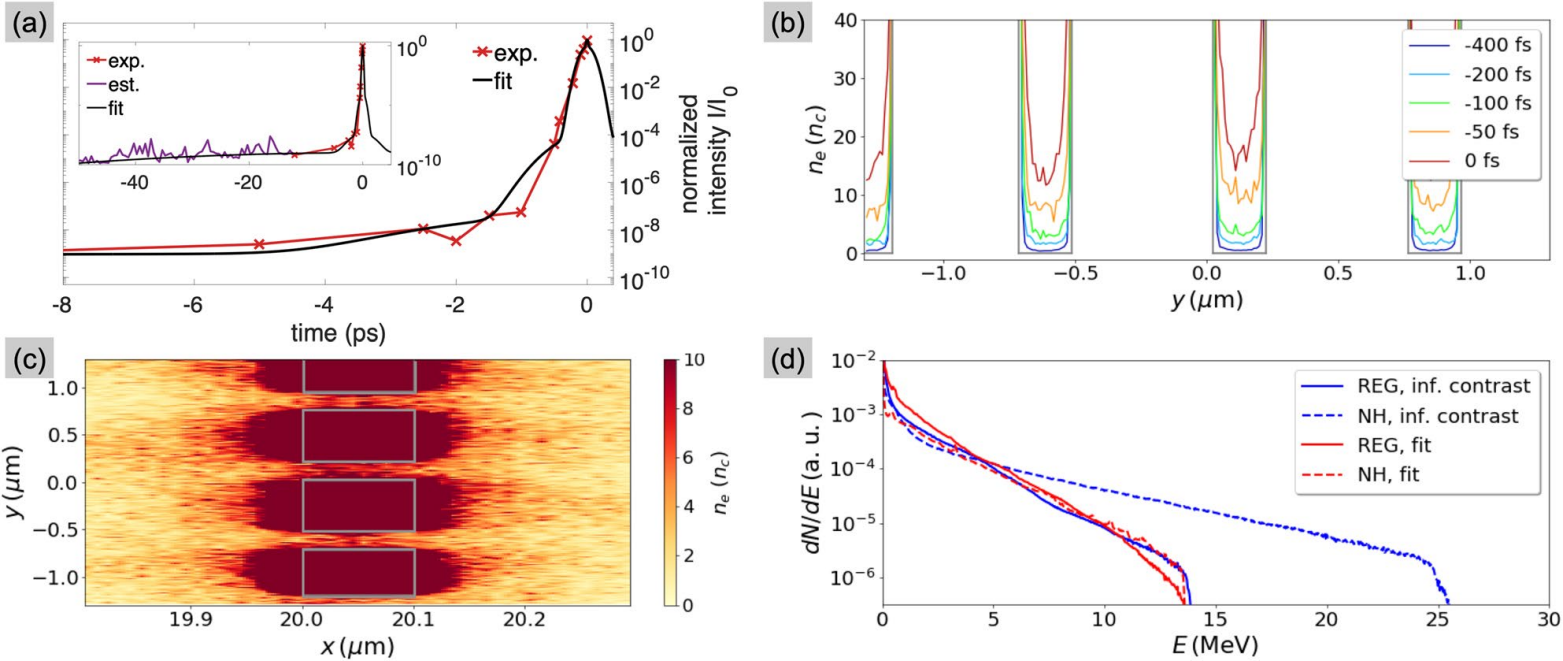
Results of the PIC simulations with finite contrast. (**a**) Profile of the laser pulse that fits the cross-correlator trace. The entire simulation setup is described in Methods. (**b**) Evolution of the plasma density inside the NHs at different times before the peak of the pulse (fixed at $t=0$ fs). The traces are obtained by integrating the density maps along the whole target thickness. An example of density map, taken at $-50$ fs, is shown in (**c**), where the grey boxes highlight the original target shape. (**d**) Comparison of proton spectra from regular foil and NH target, obtained with either the infinite-contrast or with the fitted laser pulse.

A pre-plasma with $L \sim 50$ nm $>L_\text {fill}$ thus results in filling of the NHs and suppression of the enhanced proton acceleration. It is worth noticing that such estimation for *L* depends on how accurately the pre-plasma initialised on the different target surfaces reproduces their actual two-dimensional expansion. To get around this limitation, as well as to support the presence of the pre-plasma in the experiment, we performed a simulation where we represented the laser pulse using a sum of Gaussian functions, so as to fit the experimental measurement of the temporal contrast^39,40^. The fitted temporal profile is shown in Fig. 6a.

With some laser energy irradiating the unperturbed solid target before the peak of the pulse, holes are gradually filled up by the expanding plasma. This is clearly presented in Fig. 6b, which represents the temporal evolution of the plasma density inside the NHs. Such plasma reaches the relativistic critical density $\gamma n_c=3.7n_c$ at $\sim -100$ fs, and it has become fully overdense, with $n_e \sim 15 n_c$, at the arrival of the peak. At this time, the scale length is estimated as $15n_c= 2n_{e0}\exp {[-d/(2L_\text {PIC})]}$, giving $L_\text {PIC}\simeq 25$ nm. The two-dimensional density map of the target, showing the holes filled at $-50$ fs, is presented in Fig. 6c for completeness. Fig. 6d shows the results on proton spectra: The $\times 1.8$ enhancement of the maximum energy with respect to a regular foil, expected when the NHs are preserved, is entirely lost with filled NHs.

## Discussion

Our experiment shows that the proton energies achieved with NH targets are similar to those obtained with regular foils of the same thickness. Supported by simulations, we relate this finding to the formation a short-scale length plasma on the target surface, caused by a fraction of uncompressed laser energy that irradiates the target in the last ps before the peak of the pulse. This happens because the DPM is designed to remove the energy of the ASE pedestal, but it becomes ineffective at the high fluences reached in the proximity of the peak. Such limitation rarely affects experiments on proton acceleration from thin targets, since a short-scale length plasma (with $L< \lambda /10$) is usually considered negligible to the efficiency of vacuum heating^34^. However, our work demonstrates that the observation of energetic protons from both surfaces of nm-thick foils, does not ensure that all types of nanostructures are preserved before the interaction with the relativistic laser pulse.

The simulations accounting for the finite contrast of the laser pulse allowed us to determine that $L_\text {PIC} \simeq 25$ nm is detrimental for the energy enhancement expected with NHs of 200 nm diameter. Since we did not measure the plasma scale length produced in the experiment, we now compare $L_\text {PIC}$ with analytical estimations of the target expansion.

The plasma scale length is calculated as $L_\text {exp}= c_s t$, with *t* the expansion time and $c_s[$nm/ps$]~=310(ZT_{e,[\text {keV}]}/A)^{1/2}$ the sound speed for the ion with charge *Z*, atomic mass *A*, and electron temperature $T_e$. To simplify our analysis, we focus our attention on the layer of hydrogen contaminants present on the target surface and on the NH walls, because the protons dominate the dynamics of the plasma in the early stages of its expansion, when the number density of protons is higher than the number density of gold ions, $n_p> n_{\text {Au}^+}$^41^. We expect $L_\text {exp}^{\text {H}^+}$ to give an overestimation of the scale length actually produced in the experiment, because the formula assumes that the expansion is sustained by a semi-infinite plasma^36^, whereas recent measurements^42^ limit the the thickness of the contaminant layers on metallic surfaces to 1–5 nm $< L_\text {PIC}$. The expansion of the gold target is therefore essential to produce the plasma that fills the holes. However, a proper estimation of the plasma scale length requires sophisticated assumptions about the average ionisation of the foil in the moments before the interaction with the ultrashort pulse. In the following, we calculate $L_\text {exp}$ both for the hydrogen plasma produced from the contaminant layer, and for the gold plasma formed by 11-time ionised atoms. This value is chosen^43^ considering the charge state of a gold atom caused by field ionisation at a laser intensity of $\sim 10^{15}$ W/cm$^2$. We reckon that the experimental scale length will be within the range determined by $L_\text {exp}^{\text {H}^+}$ and $L_\text {exp}^{\text {Au}^{11+}}$ .

The electron temperature is given by^36^ $T_e[$eV$]~=119 (10^{-23}n_{e0,[\text {cm}^{-3}]})^{1/12} Z^{1/12} (10^{-15}I_{\text {[W/cm}^2]})^{1/3} (10^{-2} \tau _\text {[fs]})^{1/6}$, and it depends on the intensity *I* that causes the heating during the time span $\tau$. Since the target is irradiated by the ps-shoulder of the pulse, the heating time and the expansion time are considered equivalent, $t=\tau$, and we take the intensity arriving on target at $-500$ fs as the starting point for our calculation. This is motivated by two reasons: First, the contrast measurement in Fig. 1b clearly shows that at this time the DPM is ineffective; Second, even if target ionisation occurs at a lower intensity (i.e. before $-500$ fs), electrons heated at later times will catch up with the earlier, slower expanding front, because of their higher temperature. Taking $10^{13}$ W/cm$^2$ as lowest threshold for target ionisation, $I=2 \times 10^{15}$ W/cm$^2$ at $\tau = -500$ fs represents the mid-point for calculating the expansion velocity during the irradiation by the ps-long shoulder.

For the initial electron density we use respectively the number of ionised hydrogen atoms in water, $n_{e0}^{\text {H}^+}= 6.7 \times 10^{22}$ cm$^{-3} \simeq 40 n_c$, and the electron density of the ionised gold, $n_{e0}^{\text {Au}^{11+}}= 6.5 \times 10^{23}$ cm$^{-3} \simeq 370 n_c$. For the hydrogen plasma, it follows that $T_e^{\text {H}^+}=190$ eV, $c_s^{\text {H}^+}=135$ nm/ps and $L_\text {exp}^{\text {H}^+} \simeq 70$ nm. For the gold plasma, $T_e^{\text {Au}^{11+}}=280$ eV, $c_s^{\text {Au}^{11+}} = 40$ nm/ps and $L_\text {exp}^{\text {Au}^{11+}} \simeq 20$ nm. We also notice that with the electron temperature rapidly increasing in the last 500 fs, gold atoms with higher ionisation states would have higher sound velocities and produce larger scale lengths.

Despite the order of $L_\text {exp}$ is below $\lambda /10$, the estimated scale lengths are crucial in relation to the size of the NHs. Compared to $L_\text {PIC}$ and $L_\text {fill}$, the results for $L_\text {exp}$ support our interpretation that the plasma produced by the ps-long shoulder fills the NHs before the interaction with the ultrashort laser pulse. The overdense plasma removes the additional interaction surface that was made available with the NH walls, and it prevents the electromagnetic field of the incoming pulse from recirculating the electrons around the holes. The result is a reduction both of the number and of the energy of the electrons accelerated during the interaction, hence the suppression of the mechanism for enhanced heating. The experimental results indicate that the filling occurs also with larger NHs, allowing us to infer that $L_\text {exp} \simeq 50$ nm, which is the value for $L_\text {fill}$ when $d=510$ nm. We can also notice that $L_\text {exp} \propto I^{1/6}$, so reducing the intensity on the target by a factor of 100, as done in the experiment by moving away from the laser focus, only decreases the scale length by a factor of 2, which is not enough to prevent the filling of the NHs.

## Conclusion

In this work, we tested the efficiency on proton acceleration of ultrathin flat foils perforated with non-periodic distributions of holes of nanometric size. According to numerical simulations with laser pulses with infinite temporal contrast, electrons extracted from the the walls of the NHs and accelerated in the interstices lead to enhanced accelerating fields and high proton energies. However, in our experiment we could not observe any significant difference between the energies measured with NH targets or with regular flat foils. Our explanation, supported by simple analytical estimations and by PIC simulations, is that the laser temporal contrast obtained with a standard double plasma mirror does not prevent the formation of a short-scale plasma that fills the holes prior to the interaction with the relativistically intense laser pulse. It is worth emphasising that such a plasma does not particularly alter the efficiency of vacuum-heating, hence resulting in proton energies of similar magnitude from both surfaces of the foils. In this sense, measuring proton acceleration from the front surface does not allow discriminating the finer conditions of the target surface.

Despite the fact that NH targets do not lead to enhanced proton energies, experimental investigations such as this one remain crucial to assess realistic interaction conditions and to point out the limitations of present target and laser technologies. The study of the heating mechanisms and absorption processes enabled by suitable nanostructures continues to be relevant to identify the key parameters that improve proton acceleration. But there is further need for target fabrication techniques and laser development to progress hand in hand, as the contrast typically achieved in multi-TW laser installations may yet be a limit for the capabilities of advanced target geometries. As of now, the laser community is striving to deliver PW-class systems with unprecedented contrast both in the ns and in the ps timescale^44–47^ , exploiting different techniques to maintain clean pulses from the laser front end, through the amplification chain, and up to plasma mirrors^48^. At the same time, monitoring of on-target plasma formation, with sub-ps temporal resolution, will help correlating the shot-to-shot conditions of the target surface with the results from proton diagnostics, and modelling accurate experimental conditions in the numerical simulations. Further studies of the interplay between target geometry and laser properties will finally open the way to new strategies for the optimisation of proton acceleration, both in the TW and in the PW regime.

## Methods

### Target fabrication

Ultrathin gold foils with nanoholes (NHs) were manufactured at the Department of Physics of Gothenburg University, using hole-mask colloidal lithography^27^. The main steps of the fabrication are: (1) deposition of a sacrificial layer of Cr (70 nm thick) on a clean glass substrate; (2) deposition of a colloidal solution, containing polystyrene nanospheres with diameter equal to the required nanohole size; (3) deposition of the Au film, with thickness smaller than the nanospheres diameter; (4) tape-stripping of the nanospheres; (5) etching away the Cr layer, rinsing in distilled water and (6) transfer of the perforated film onto the target holder.

This fast and versatile technique allowed to prepare NH targets with different combinations of hole diameter ($d= 230$, 510 nm), filling factor ($\rho = 5$, 15, $25\%$) and substrate thickness ($t=120$, 250 nm). The reference Au foils, without holes, were fabricated in the same deposition cycle as the NH targets, by simply skipping the deposition of the nanospheres.

All targets were checked and characterised with scanning electron microscopy after transfer onto the target holder (see Fig. 1). This one, in particular, fitted up to 4 different target types at the same time, allowing to compare their performances in the same experimental cycle.

### Experimental setup

The experiment was carried out at the High Power Laser Facility of Lund University (Sweden), whose Ti:Sapphire laser system delivers 35 fs pulses at a central wavelength of 0.8 $\upmu$m. The Gaussian, P-polarised beam was focused on solid targets by an *f*/3 off-axis parabolic mirror, within a $\sim 2.5$ $\upmu$m FWHM focal spot. With an incidence angle of $45^\circ$ and a maximum energy of 350 mJ, the peak intensity on target reached $I \simeq 10^{20 }$ W/cm$^2$.

A double plasma mirror^32^ (DPM) allowed irradiating the ultrathin metallic foils without reaching their ionisation threshold ($\sim 10^{13}$ W/cm$^2$ for Au) with the 100s of ps-long pedestal due to amplified spontaneous emission (ASE). The DPM consists of two glass substrates with an anti-reflective coating, arranged with two parabolic mirrors in a confocal setup. The beam is transmitted through the substrates until the energy deposited in the material is enough to trigger a ionisation cascade. When the electron density finally reaches the critical value at the laser wavelength, the beam is reflected to the re-collimating mirror and transported to the experimental chamber. The fluence of the incoming beam on the dielectric substrates determines the trigger time of the device. A measurement of the laser temporal contrast, obtained with a third-order cross-correlator, is presented in Fig. 1b. Without DPM, the level of the ASE pedestal (at $\ll -100$ ps) is about $10^{-9}$ times the intensity of the peak of the pulse, while it falls outside the dynamic range of the cross-correlator when the DPM is used. In addition, a contrast enhancement of $\sim 3$ orders of magnitude is achieved at $-2$ ps, thus preventing target ionisation until $\sim 1$ ps before the peak.

Targets were mounted and aligned under vacuum at the position of the laser focus with $\sim 10$ $\upmu$m accuracy, and the quality of the irradiation site was verified prior to each shot. During the experiment, we also moved the targets along the laser focal axis, in order to illuminate larger areas of the foils without reducing the laser energy delivered both to the DPM and to the target. Given our Rayleigh length $z_\text {R} \sim 18$ $\upmu$m, shifting the target by 50, 100 and 200 $\upmu$m from the focal plane corresponded to approximately a 3, 6 and 10-time increase of the focal spot radius, hence to lowering the intensity by a factor of 9, 36 and 100.

We measured the energy spectra of the proton beams emitted along the normal direction to both surfaces of the target, front and rear. Protons accelerated from the front, laser-irradiated surface (backward direction, BWD) were detected by a calibrated magnetic spectrometer. The dispersed particles impinged on a scintillator (St. Gobain, BC-408) protected by 12.5 $\upmu$m of Al foil to filter out spurious light and heavier ions. Protons accelerated from the rear surface of the target (forward direction, FWD), instead, were measured with a calibrated Thomson Parabola spectrometer, employing a micro-channel plate and a phosphor screen as detector.

We also used two Spectralon screens (LabSphere, SRT-99-050) to intercept the laser light that was reflected and transmitted by the target. To prevent damage, the Spectralons were placed at $\sim 15$ cm distance from the target. An image of each screen was recorded by a dedicated CCD camera provided with neutral-density and 800-nm interferometric filters. The integrated signal was rescaled to take into account all the attenuation factors and different collection angles of the cameras. Then, we compared the signal obtained when irradiating different targets to the one recorded on the transmission Spectralon when no target was in place (blank shots).

### Particle-in-cell simulations

Particle-In-Cell (PIC) simulations were performed with the two-dimensional version of the Smilei Code^49^, adapting the numerical setup originally published in Ferri et al. (2020) ^26^.

We used a P-polarised laser pulse, with $0.8~\upmu$m wavelength and Gaussian profiles both in space and time, characterised by a FWHM of 2.5 $\upmu$m and 35  fs, respectively. The energy of 350 mJ corresponded to a normalised potential vector $a_0 = 6.7$ at the centre of the spot. The pulse was focused at $45^\circ$ of incidence on 100 nm-thick gold foils (11 times ionised, with ion number density $n_{\text {Au}^{+}}= 5.85 \times 10^{22}$ cm$^{-3}$, 100 macro-particles per cell, per species), coated on both surfaces with a 10 nm-thick proton/electron layer ($n_{p}= n_e= 1.74 \times 10^{23}$ cm$^{-3}$, 1000 macro-particles per cell, per species). For the perforated foils, holes with 200 nm diameter and $30\%$ filling factor were randomly distributed on the target surface, following the method described in Ferri et al. ^26^ The simulation box was $50\times 56~\upmu$m$^2$, with spatial steps $\Delta x = \Delta y = 5~$ nm and a time step $\Delta t = 11.7$ as.

In the simulations with the pre-formed plasma, the proton/electron layers were given an exponential density profile of the form $n_e=n_{e0}\exp {(-s/L)}$, *s* being the distance from the unperturbed target (with $n_{e0}=11 n_{\text {Au}^{+}} \simeq 370 n_c$) and *L* the scale length (varied between 0 and 70 nm). The profiles, added to all target surfaces (front, rear, and NH walls) were cut where $n_e=0.01~n_c$ to limit the spatial extent of the pre-plasma. The number of macro-particles in the pre-plasma was increased to 200 when working with $L=10$ nm.

To account for the finite temporal contrast measured in the experiment, we modelled the intensity profile of the laser pulse with a sum of Gaussian functions, so as to describe the ultra-short pulse, the ps shoulder and the earlier ASE pedestal^39,40^. The normalised amplitude *A* and the width $\sigma$ (in ps) for each function $A\exp {[-t^2/(2\sigma ^2)]}$ were chosen so that the third-order cross-correlator trace would fit the experimental measurement up to 50 ps before the pulse peak, as shown in Fig. 6b. In detail, $A=0.5$ and $\sigma =0.015$ ps for the ultra-short pulse (such that FWHM$=2\sigma \sqrt{2\log (2)} = 35$ fs), $~A=0.5$ and $\sigma =0.09$ ps for the shoulder between $-500$ fs and $-1$ ps, $~A=10^{-4}$ and $\sigma =0.35$ ps for the shoulder between $-1$ fs and $-2$ ps, $A=4 \times 10^{-8}$ and $\sigma =1.5$ ps for the pedestal between $-5$ and $-2$ ps, and $A=10^{-9}$ and $\sigma =25$ ps for the pedestal before $-5$ ps. With this profile, the transverse size of the simulation box was increased to $92~\upmu$m. We also decreased the peak intensity in order to redistribute the same 350 mJ of laser energy along such a longer pulse. A scaling factor of 3.5 was calculated as the ratio of the areas subtended by the fitted intensity profile and by the ultrashort pulse. As a consequence, $a_0 = 6.7/\sqrt{3.5} = 3.6$. The simulations run from 1 ps before the peak of the pulse, to $t=250$ fs, when the proton spectra were recorded. Irradiating the targets with the longer laser pulse caused heating and expansion of the foil, for both target types. The expansion of the rear surface, in particular, motivated adding the pre-plasma also on the back of the target, when varying the pre-plasma scale lengths.
